# Supplementary material for: Patient-provider relationships in China: A qualitative study on the perspectives of healthcare students and junior professionals
Source: PLoS One. 2020 Oct 21;15(10):e0240747. doi: 10.1371/journal.pone.0240747 (PMC7577488; doi:10.1371/journal.pone.0240747)
Supplement: Attachment — Submitted filename: Response Letter PONE-D-20-17587 2020AUG10.docx [file pone.0240747.s001.docx]

Dear Dr. Zhang,

We appreciate the opportunity to address the comments and revise our manuscript. Below, please find item-by-item responses to the comments.

**Manuscript Title:**

Patient-Provider Relationships in China: A Qualitative Study on the Perspectives of Healthcare Students & Junior Professionals

***Authors:*** Yuxian Du, Ph.D.; Yan Du, Ph.D., MPH, RN; Nengliang (Aaron) Yao, Ph.D.

| Reviewer # | Manuscript Location | Quote of Comment (if any) | Authors’ Response |
| --- | --- | --- | --- |
| Journal Requirement | Everywhere | “Please ensure that your manuscript meets PLOS ONE's style requirements, including those for file naming.” | Revised accordingly. Thanks! |
| Journal Requirement | Data availability | “We note that you have indicated that data from this study are available upon request. PLOS only allows data to be available upon request if there are legal or ethical restrictions on sharing data publicly.” | We don’t have the participant’s consent to release the original data. |
| Journal Requirement | Article information | Update (a) Funding Statement; (b) Competing Interests Statement | We have updated both the Funding Statement and Competing Interests Statement to include information as requested. As the guideline instructed, these changes will be submitted through the online submission system. |
| Journal Requirement | Results | “Please ensure that you refer to Tables 1 and 2 in your text” | These two tables are now refereed on page 8, lines 149 and page 9, line 160. |
| Editor | Methods | “This interesting study addresses a critical problem in China, but there are some methodological concerns. Please be mindful that unsatisfactory responses to the comments from reviewers and the editor may result in rejection to the revision. In other words, the chance of accepting the revised manuscript is very small unless very significant changes are made.” | We appreciate comments from you and the peer reviewers. We have addressed all the comments and made significant changes, ensuring accuracy and robustness of the methodology. |
| Editor | Methods | Major Points: “1. The sample size is small, which mandate smaller/fewer subgrouping. Thus, Table 1 may need to re-group some of the categories.” | Thanks! We have updated Table 1 so that some categories are regrouped together to avoid small numbers in many groups. The only exception in category “others/unknown” in the variable “sector”, because it would not be reasonable to combine this category with either of the remaining two categories. |
| Editor | Methods | Major Points: “2. Fisher exact test should be used in Table 1 since some cells have a number smaller than 5.” | We have updated Table 1 so that the p-values are all from Fisher exact test. |
| Editor | Methods | Major Points: “3. Please provide how many people received or read the advertisement so that we will better understand the potential selection bias.” | Thanks for the question. Honestly, we don’t know how many people have seen the poster. We have added this as a limitation (page 18, lines 345-346). |
| Editor | Methods | Major Points: “4. Were the training free? Were the participants compensated or provided other incentives?” | The training was free to healthcare students. Junior healthcare professionals paid 2000CNY for registration. This information is added to the first paragraph of the methods section (page 5, lines 102-104). |
| Editor | Methods | Major Points: “5. In my understanding, there were only 13 participants who answered the questionnaires as shown by researcher 1. The sample number is very small. Further, if it is true, only about 8.5% (13/151) participants responded. The proportion of missing data appeared too big.” | It is a great question. Researcher 1 used a “coding until saturation” method, which did not use all the survey responses. That’s why the overall counts of codes are different between Researcher 1 and 2. We have added a note under Table 2. |
| Editor | Tables | Minor points: “1. In tables, please provide a note indicating ref=reference.” | We have added notes in Table 3-5 indicating that “ref = reference”. In addition, we explain our criteria in counting appearance of themes. |
| Editor | Methods | Minor points: “2. Please indicate how many participants answered the questionnaire about patient physician relationship.” | The “coding until saturation” method in Table 2 can be a little confusing to readers that are not familiar with qualitative analysis. All 189 participants answered the question about patient-provider relationships. Researcher 1 used a “coding until saturation” method, which did not use all the survey responses. And Researcher 2 used all responses. |
| Reviewer #1 | Methods | “1. There are 151 healthcare students and 38 junior professionals from 20 provinces in China. What is the representativeness of sample? Is there difference of Patient-Provider relationships between healthcare students and junior professionals?” | Thanks for the comment. The “representativeness” in quantitative research is often replaced with “saturation” in qualitative studies. Saturation is a criterion for discontinuing data collection and/or analysis. For example, you can hear me talking for a day, but I may repeat lots of points during the speech. It reaches the saturation point when you find the talk is boring and repeating itself again and again. In our study, views from junior professionals and healthcare students reached saturation at sample sizes of 4 and 16. In other words, our findings are “representative” of views of those two groups. We have added a sentence in the methods section. |
| Reviewer #1 | Methods | “2. Geographical Region in Table 1 is “east,south and north”. What is the basis of regional division? In general, the region is divided into “east,central and west” or “south,central and north”.” | We illustrated our basis of regional division and explained why we combined some regions into larger categories. Details are on page 7, lines 134 – 143. |
| Reviewer #2 | Introduction | “1. Some statements in Introduction need to be supported by more recent data, such as in line 6, the first paragraph in Introduction, literature 7.” | Thanks for the reminder. We were able to find more recent data and updated the citations (page 4, lines 67-68). |
| Reviewer #2 | Introduction | “2. The second paragraph in Introduction need to be more detailed about why patients-provider relationship is relatively serious.” | We have added two sentences to describe of the significance of this study (page 4, lines 78-80). |
| Reviewer #2 | Everywhere | “3. Some words in this article is not spelling correctly.” | Thanks for this comment. We have carefully checked throughout the manuscript and ensured all words are spelled correctly. |
| Reviewer #2 | Methods OR Discussion | “4. In my opinion, health care provider should be workers in hospital or health institution. However, respondents in this study are most students. The views of healthcare professional on PPR are different from those of students, personally thinking. Why did the author think the opinion of students could represent patient-provider relationship?” | Thanks for the questions. We were not trying to “measure” the patient-provider relationship. Instead, we wanted to give a voice to healthcare students and junior professionals. This qualitative study presents the opinion from the healthcare students and junior professional as a unique perspective per se and would not state that their opinion could represent provider or the patients in the patient-provider relationships.  We mentioned and highlighted this in the revised manuscript (pages 9-10, lines 176-178). We also add this limitation in the revised manuscript (page 19, lines 359-363). |
| Reviewer #2 | Methods | “5. The professional quality of two researchers need to be illustrated in Method.” | We appreciate the comment. We have added a description in the 2^nd^ paragraph of methods, on page 6, lines 113-115. |
| Reviewer #2 | Methods | “6. The Patient’s influence is stilled answered by Providers. Answer from patients may be more reliable, considering the focus of this article is patient-provider relationship.” | Thank you for this comment. We acknowledge the fact that “the patients’ influence” was answered by healthcare students and junior professionals”.  We mentioned and highlighted this in the revised manuscript (pages 9-10, lines 176-178). We also add this limitation in the revised manuscript (page 19, lines 359-363). |
